# Evaluating massage therapy for radiation-induced fibrosis in rats: preliminary findings and palpation results

**DOI:** 10.1080/15384047.2024.2436694

**Published:** 2024-12-02

**Authors:** Geoffrey M. Bove, Holly McMillan, Mary F. Barbe

**Affiliations:** a Owner and Principal Investigator, Bove Consulting, Kennebunkport, ME, USA; b Department of Head and Neck Surgery, Senior Speech Pathologist and Clinical Research Fellow, Texas School of Public Health, University of Texas MD Anderson Cancer Center, Houston, TX, USA; c Aging + Cardiovascular Discovery Center, Lewis Katz School of Medicine, Temple University, Philadelphia, PA, USA

**Keywords:** Musculoskeletal manipulations, radiotherapy, post-exposure prophlaxis, brachial plexus neuropathies, physical examination

## Abstract

Radiation-induced fibrosis (RIF) is a common side effect of cancer treatment, but can manifest into a devastating syndrome for which there is no preventive measure or cure. In rats who perform a repetitive work task, who left untreated develop signs and symptoms that resemble repetitive motion disorders in humans, we have shown that manual therapy prevents the development of fibrosis and other key biomarkers. The fibrosis of RIF and repetitive motion disorders has similar biomarkers. In rats, we sought to determine if manual therapy would alter key biomarkers of post-irradiation fibrosis following X-ray irradiation given to the rat forelimb. One limb of rats was given a damaging dose of X-ray irradiation. Some limbs were massaged using a protocol previously described and characterized. Serum inflammatory markers, histological assays of tissue fibrosis and nerve pathology, and electrophysiology for neuropathic discharge were assayed after 8 weeks. We also tested if an experienced therapist could identify the irradiated limb using blinded palpation at the 8 week end-point. While preliminary assays showed robust changes compared to control limbs, the other assays did not show similar pathology. Our therapist could detect each irradiated limb. Serum inflammatory markers were reduced by massage to the irradiated limb. We conclude that blinded palpation is sensitive to detect subtle changes in tissue following irradiation. In contrast to the preliminary studies, the dose of irradiation used was insufficient to induce long-lasting deep fibrosis or nerve degeneration. We suspect that a difference in housing, and thus physical activity, was the plausible reason for this difference.

## Introduction

Therapeutic irradiation, or radiotherapy, is commonly used to treat primary cancers and areas of suspected metastasis. Despite improvements in targeting and dose optimization, radiation-induced fibrosis (RIF) can lead to radiation fibrosis syndrome (the clinical and often symptomatic manifestation of RIF). While likely to occur to some degree following all treatments, RIF becomes clinically relevant to more than >75% of patients, in a highly tissue-dependent manner.^1–4^ Radiation-induced fibrosis can cause adverse side effects attributed to pathology of the neuromuscular structures, including radiating pain and paresthesia, motor limitations, and altered function.^5,6^ Early effects include muscle and vascular damage leading to capillary network failure and ischemia,^6–8^ and endothelial-to-mesenchymal cell transition to collagen producing cell types via activation of TGFβ-1 downstream signaling.^9^ Late effects of radiotherapy are dominated by the continued and insidious accumulation of collagen within muscles, nerves, and vessels,^5,6,8,10–12^ and neuropathies of radiation-exposed nerves.^13–18^ Human and animal model biopsies show that a key culprit of progressive radiation-induced neuropathy is the development of extraneural and intraneural fibrosis (nerve and axonal entrapment), fibrous replacement of axons, demyelination, and damage of the neurovasculature.^8,19,20^ Part of the difficulty with treatment is that once extracellular matrix is cross-linked, it becomes resistant to the degradative breakdown needed for restorative repair.^21–23^

In a rat model of overuse, a progressive fibrosis develops and causes functional disabilities, and histological, biochemical, and electrophysiological indices of muscle and nerve pathology.^24–31^ Several underlying mechanisms driving fibrosis appear to be fundamentally similar between the two etiologies, such as the TFG-ß mediated response of fibroblasts (reviewed by Fijardo et al.)^3^ It follows that the treatments that are effective in the overuse model^24–26,29,30,32^ should be effective for prevention of RIF. The most effective treatment to date has been manual therapy.^29,33,34^ We therefore hypothesized that manual therapy could be an effective preventive of RIF. We also hypothesized that post-irradiation soft tissue change could be detectable by palpation. Here, we report the results of our initial investigations addressing this hypothesis.

## Results

### General

Please see the Supplemental File for the preliminary results that informed the design of the study described herein, as well as the overall conclusions. All rats survived irradiation and transport. No rat displayed any signs of illness during the experiment, and all gained weight normally. As in the preliminary studies with rats given primarily a 10 Gy dose of irradiation, some rats given a 15 Gy dose showed transient hair loss on the anterior distal forelimb with full recovery by 8 weeks post-irradiation (see Supplemental File).

### Responses during treatment

As in our previous studies, the rats accepted the treatments, their behavior varying more by the individual rat than by the irradiation side. Between treatment weeks 2 and 4, 4 of the 32 rats exhibited behavior consistent with tenderness, namely withdrawal of the limb from the treater’s grasp when forelimb muscles were being targeted. In these rats, the treatment remained the same, but the pressure was limited to the threshold of the withdrawal. The tenderness did not remain past 4 weeks. There was no aggressive behavior of these or the other rats in response to the treatment or otherwise.

### Identification of the irradiated limb by blinded palpation

In the forced choice task, HM identified the irradiated limb in each of the 16 rats (100% accuracy). Her judgment of fibrosis in the non-irradiated limb was always 0 (none), and her judgment of fibrosis in the irradiated limb ranged from 0 to 2 (none to mild) (mean 1.125 ± 0.8 (SD)). This difference was statistically significantly different (*p* = .0005 ± 2.0 (CI). Her confidence in her choice ranged from 3 to 5 (mean 4.1 ± 0.8 (SD)). She consistently referred to the irradiated limb without manual therapy as either “dense,” smaller,” or “atrophied” (8/8 irradiated, non-treated limbs). Of the limbs that were irradiated and treated with manual therapy, 4/4 were identified as “bigger” or “more columnar” (right limbs); 4/4 (left limbs) were not confidently qualified, with comments such as “tough to tell,” “maybe atrophied,” “a little atrophied,” and “similar,” but were still accurately identified as irradiated. This qualitative analysis suggests that the therapist was identifying some difference due to the manual therapy of the irradiated limbs, and that this made it more difficult to discern between the limbs on palpation.

### Identification of the treated limb

Rats were presented to HM with the knowledge of which limb was irradiated, to choose whether the limb had been treated with manual therapy. The therapist was not able to reliably discern differences between irradiated and untreated versus irradiated and treated limbs (4 correct and 12 incorrect choices).

### Inflammatory mediator assays

**Figure 1.**
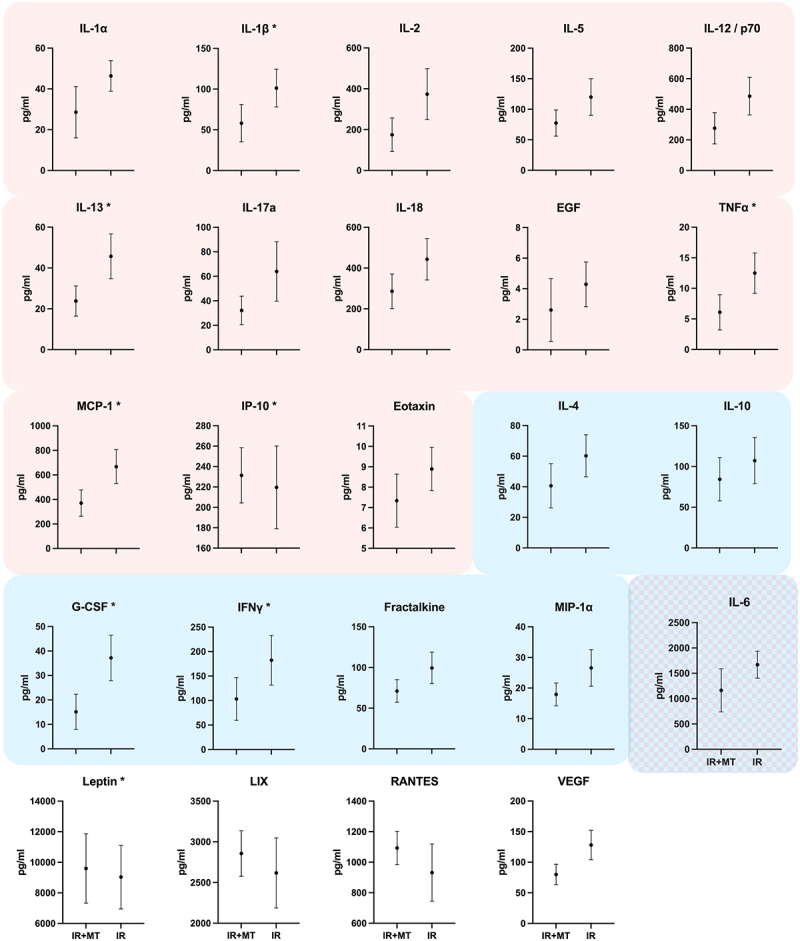
Serum levels of cytokines and chemokines of irradiated rats who had their irradiated limb massaged (IR-MT) or not (IR). (Figure was patterned after Bove & Mokler^35^, where an overview of the functions of the substances assayed can be found.^35^ Pink and blue shades indicate that the substances have been reported to be inflammatory or anti-inflammatory, respectively. There was a trend for the majority of the substances to be decreased in rats that had their irradiated limbs massaged, however, there were no statistically significant results (t-tests).

None of the assayed inflammatory mediators were statistically significantly different between the groups analyzed. However, there was a trend in that the rats who had their irradiated forelimbs massaged had lower mediator levels in most of the assays (Figure 1).

### Collagen content (fibrosis) of the muscles and neurovascular structures

**Figure 2.**
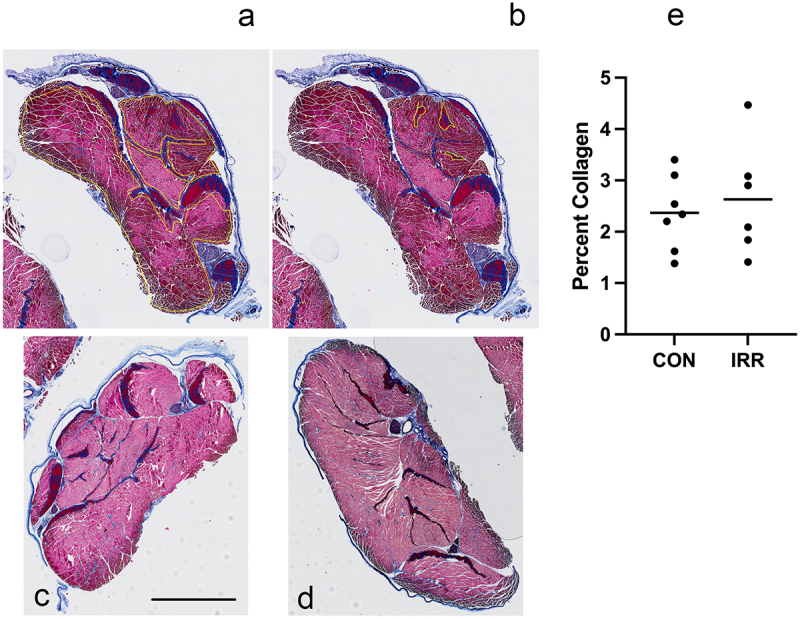
Trichrome staining and method for quantification of collagen. a. Regions of interest (ROIs) outlined in yellow. Collagen is stained blue. The amount of blue was quantified. b. in the same section, ROIs to be excluded are outlined in yellow. c and d. Representative images of sections from control (unirradiated) and irradiated and untreated limbs, respectively. e. Quantification of control and irradiated showed no difference between groups. Scale bar (in c) = 100 µm.

In stark contrast to our preliminary experiments, there was no apparent increase in collagen (indicating fibrosis) due to the irradiation when comparing non-irradiated limbs to irradiated limbs (Figure 2c–d). Quantification of these two groups reflected this lack of difference (Figure 2e); thus, there was no difference possible to detect due to the treatment. The collagen percentages (1–4%) are consistent with control percentages from previous studies.^36,37^ Nerve sheath and major blood vessel wall thickness were comparable in all sections. Picrosirius red stained sections did not reveal any differences between irradiated and non-irradiated limbs.

### Evidence of active musculoskeletal inflammation

While our present method showed robust labeling in spleen sections, there was no ED-1 labeling in musculotendinous structures of the irradiated limbs. Therefore, there was no detectable difference between the treated and untreated limbs.

### Neuropathology

**Figure 3.**
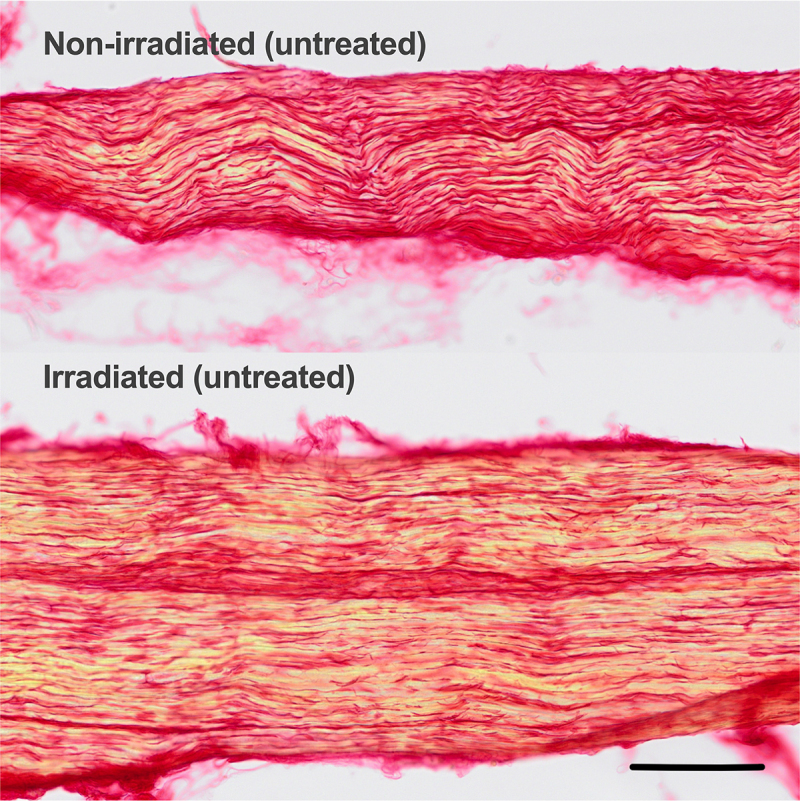
Picrosirius staining for collagen in nerve. Collagen is stained yellow in this method. Representative images of control (top) and irradiated (bottom) nerves are shown. When quantified, there was no difference between groups. Scale bar = 100 µm.

Electrophysiology was performed on 4 irradiated limbs. Forty-nine neurons with conduction velocities identified in the C- and slow A-delta range were recorded. None of these neurons had ongoing activity and were therefore judged as normal. Sixty neurons with faster conduction velocities were recorded in these same experiments. These neurons were a mix of cutaneous and deeper low threshold mechanoreceptors, and muscle spindles. There was no discharge consistent with pathology, as we have previously described.^29,38^ Since no pathological discharge was recorded in these experiments, recordings from the irradiated and treated limbs, and the non-irradiated limbs, were not performed. Instead, the median nerves from these rats were harvested and processed for collagen content (fibrosis), and myelin degeneration. Nerve picrosirius red staining was performed to compare collagen between untreated irradiated limbs and non-irradiated limbs. There was no visually detectable difference in collagen due to the irradiation (Figure 3). Percent area collagen in the nerves within irradiated and non-irradiated groups ranged from 9% to 25%, and did not differ between groups. Because there was no difference because of the irradiation, there was no assay for detection of a treatment effect. While brain sections labeled strongly for dMBP, median nerves from irradiated limbs did not label positive for dMBP in any quantifiable level (data not shown), suggesting that any damage due to irradiation had been resolved.

## Discussion

While the inflammatory mediator panel suggested that the massage therapy lowered inflammation levels in the rats where the irradiated limb was massaged, we have little other data to support why key indicators of pathology in this group of rats seemed to heal, whereas previous rats given a smaller single dose of irradiation (10 Gy) using the exact same procedures demonstrated robust pathologies at 1–5 months post-irradiation (see Supplemental File). The only difference in the rats was their housing. Although rats at both institutions were housed in accord with the highest standards, the rats at Bove Consulting were housed in groups of 4, with 3” PVC tunnels connecting the two enclosures in a U-shape. These tubes were intended to promote movement, and our observations supported this intention. Although we do not have activity monitoring data, previous reports support that rats housed together are more active,^39^ consistent with our years of observation. However, such reports are limited, and do not show dramatic differences, let alone information regarding specific movements such as the number of hand and wrist flexions and extensions. The manual therapy treatment provided was given to promote normal movements of the soft tissues. It is possible that increased movements of the limbs through increased activity led to faster resolution of most of the injuries caused by the irradiation. This possibility is in general support of our primary hypothesis, if one considers that the effects of manual therapy on muscle and other soft tissues may be primarily due to promoting normal tissue mobilities. This is a key topic for future research.

The strongest result of this study is that using strictly blinded palpation, HM was able to accurately discern every irradiated limb. Based on our data we can conclude that it was neither deep fibrosis, nor deep inflammation. The finding supports a difference between the irradiated and non-irradiated limbs that was not assayed. It seems likely that HM was palpating increased fluid content within the muscles and other soft tissues. This could have been increased interstitial fluid (edema) or lymphatic fluid (lymphedema), which were not assayed in this experiment. It is possible what we consider a general change in immune-related factors (Figure 1 and Bove & Mokler, 2022)^35^ led to a more subtle yet palpable change. Of the non-irradiated limbs, she judged fibrosis to be a 0 (none) and on the irradiated limb from 0 to 2 (none to mild; mean 1.125 ± 0.8 (SD), which was consistent with the overall absence of pathology. Most importantly, the blinded, 100% accuracy supports that the therapist was identifying a palpable change, constituting the first such report, with or without a correlating pathology.

There are interesting and real biases regarding the palpation results. HM is an expert in the field of manual therapy in an oncologic setting, with advanced training and specific expertise in palpation and treatment of irradiated soft tissue. Because of her experiences with the pathophysiological timeline of fibrosis development in irradiated humans, HM was biased against finding deep fibrosis, although the timeline is unlikely to apply to rats. GB was biased toward finding deep fibrosis in the irradiated limbs because of previous experience with this model, the work-related model, and the manual therapy field in general. Had we found deep fibrosis we would have inadvertently made a correlation between palpation and deep fibrosis.

The driving hypothesis of this study was that manual therapy could attenuate the long-term damage cause by X-ray irradiation. However, we are not able to make any conclusions related to this hypothesis because the assays used did not show any robust signs of damage or inflammation. This study was undertaken based on extensive preliminary results (see Supplemental File) supporting that the irradiation dose used (15 Gy) was sufficient to cause substantial increases in collagen content (fibrosis), long-lasting inflammation, and nerve degeneration. Since the machine delivering the X-ray dose was tested in the exact same manner as for the preliminary studies, and since there was some hair loss and tenderness during the treatment period, it seems safe to presume that the structures were initially damaged by the irradiation. Additionally, our therapist identified every irradiated limb by blinded palpation. We therefore conclude that the structures that were assayed had mostly but not fully recovered in the 2 months following the irradiation.

While previous studies used 90 Gy in a single dose,^37^ we found that even 20 Gy caused damage that we considered ethically unacceptable. Additionally, in the initial studies, 10 Gy caused sufficient fibrosis for our assays. Yet, 15 Gy in the present study did not lead to persistent fibrosis. Future studies will need to establish the correct radiation dose to reliably induce fibrosis within ethical limits, and consider housing parameters. The scope of this project did not allow for the performance of more experiments, or for other assays, such as histology of skin and volumetric measurements of the upper limb.^40^ Post-irradiation fibrosis is due to TFG-ß mediated proliferation of fibroblasts and their differentiation to myofibroblasts (reviewed by Fijardo et al.^3^). We have shown that manual therapy in a model of repetitive motion disorder reduces tissue and circulating TGF- ß.^34^ Future investigations of manual therapy for RIF should include assays of TGF-ß.

## Conclusion

Our findings offer evidence that an experienced therapist can perceive early tissue changes in the absence of assay-confirmed deep tissue fibrosis. This is the first study focusing on the correlations of palpation, injuries, and pathology findings in an animal model; however, additional studies are necessary prior to making a conclusion about what pathologies therapists are capable of palpating. Future preclinical studies of RIF need to refine the dose, fraction size, and volume treated needed to reliably cause fibrosis, including assays of skin and interstitial spaces, and consider activity level as a factor related to the resolution of post-irradiation fibrosis.

Although the mechanism of manual therapy and its effect on RIF is still under investigation, manual therapy is increasing in popularity as a safe and feasible treatment option for patients suffering from RIF and radiation fibrosis syndrome. Emerging data support that manual therapy is effective for post-irradiation pathologies, including trismus,^41^ multiple upper quarter symptoms secondary to breast cancer treatment,^42^ and skin fibrosis.^43^ It is hoped that other groups will undertake preclinical studies of massage and related manual therapies for the prevention of RIF.

## Methods

**Figure 4.**
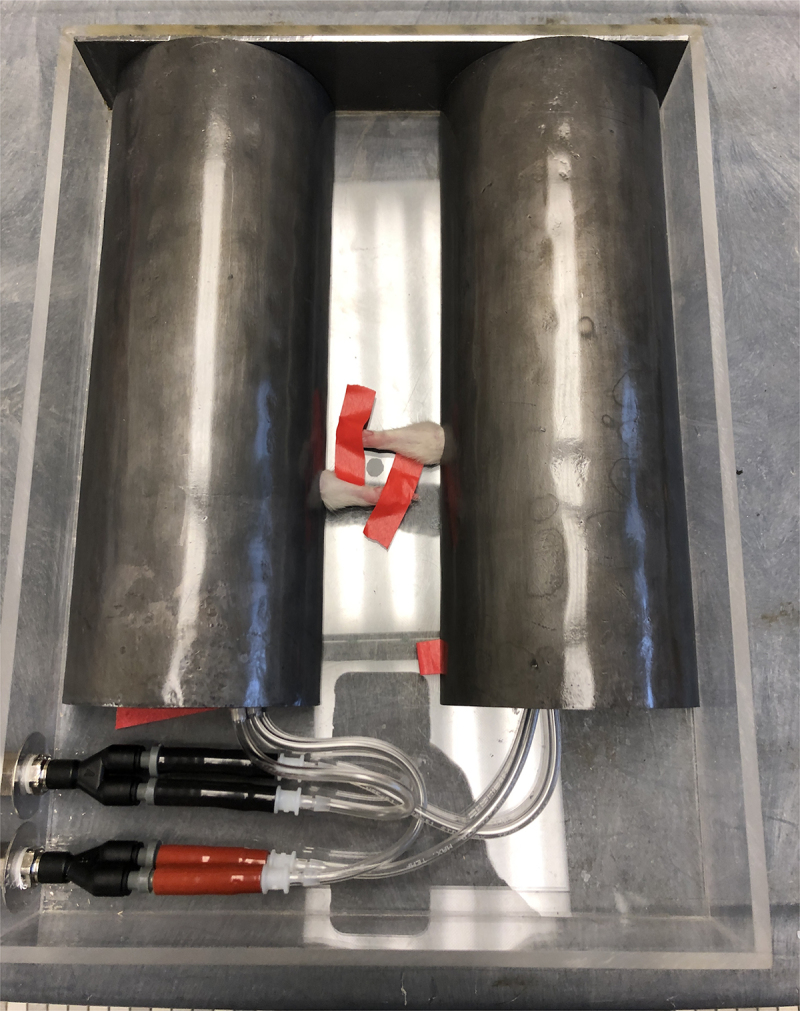
Device to shield rats from radiation. Isoflurane-anesthetized rats were positioned supine with their heads toward the operator (at the bottom of the picture). The forelimb was pulled through an opening in the side of the shield and taped for stability. A spot between the arms can be seen – this was positioned in the center of the irradiator. The anesthesia manifold can be seen at the bottom left.

Experiments were approved by the Bove Consulting and Temple University Institutional Animal Care and Use Committees in compliance with NIH guidelines for the humane care and use of laboratory animals. Studies were conducted on 42 young adult (3 months of age at onset), female, Sprague-Dawley rats (Charles River, Wilmington, MA, United States). Female rats were used to allow comparison to our past studies^23,33,38,44^ and the preliminary results of this study, which will be described as part of the current report. At both institutions, rats were fed standard rat chow and water *ad libitum*, with a 12-h light:dark cycle. At Temple University, rats were housed in separate cages with environmental enrichment in their home cages (chew toys and tunnels). At Bove Consulting, rats were housed in groups of 4, in custom cages consisting of two 18'' × 10''× 8'' tubs (Allentown, USA) connected in the back by 3'' PVC piping in a U-shape. To induce RIF, we exposed one limb of each rat to X-ray irradiation generated by an RS-2000 (Rad Source, USA). Since there is a huge range in the literature regarding the single irradiation dose needed for RIF in rats (5–90 Gray (Gy))^19,45,46^ we performed a dosing study with the goal of consistently producing neuromuscular fibrosis and sensorimotor declines while avoiding overt injury to the limb, especially the paw (see Supplemental File). We constructed a beam restriction and shielding device to limit irradiation to the elbow-fingers, connected through a manifold for isoflurane anesthesia (Figure 4). The shielding was made of 2.4 mm thick pure lead laminated to acrylic. The contralateral forelimb was shielded and not irradiated (control limb). In later experiments, the digits were shielded using ~0.8 mm aluminum foil.

To assure that we were delivering the intended dose, we used a 10X6–0.6 high Dose Rate Chamber linked to a Model 2186 control unit and Model 9660A ion chamber digitizer (Radcal, USA), placed within the irradiator. The ion chamber was placed in the center of the shelf where the rat’s forearms would be positioned, on the shielding device. We determined the duration to achieve 5, 10, 15, 20, and 25 Gy doses. The ion chamber was also placed underneath the shield, where the dose was found to be negligible (the same as background scatter within the room). We also measured the dose during an irradiation session, and found it to be accurate.

For the preliminary studies, rats anesthetized with isoflurane (4.5% in pure oxygen for induction, 2% for maintenance) were irradiated with a single dose of either 5 (*n* = 8), 10 (*n* = 8), or 20 Gy (*n* = 4). Four to 12 weeks after the irradiation, rats were euthanized and their forelimbs processed for evidence of fibrosis. Of these doses, 10 Gy consistently produced signs of inflammation and fibrosis, while 20 Gy led to unacceptable damage to the paw. For the results of these initial studies see the Supplemental File.

For the main study, the dose of 15 Gy was selected with the goal of producing more robust assays than 10 Gy without unduly harming the rats. One limb each of 32 rats was irradiated (16 rats irradiated on left limb and 16 rats irradiated on right limb). Since our preliminary experiments showed that no changes occurred to the contralateral limb, the non-irradiated contralateral upper limbs served as controls for the irradiation for each rat. The ear on the irradiated side was punched (2 mm) while the rat was anesthetized.

The day after irradiation, rats were shipped to Bove Consulting for the duration of the experiment. For practical reasons, including housing capacity and workflow, the experiment was divided into 2 groups of 16 rats. In each sub-group, there were 8 irradiated on left and 8 irradiated on right.

Starting 2 d after arrival, each rat had 1 limb treated with manual therapy. We have shown nonspecific handling is an appropriate control for this treatment,^29,34^ and that similar treatment of the lower limb does not have an effect,^33^ and so the untreated limb served as a control for the treatment. The design therefore rendered 4 experimental groups of limbs:

1. irradiated and treated
2. irradiated and not treated
3. non-irradiated and treated
4. non-irradiated and not treated

Massage therapy treatment began 3–4 d after irradiation, and was given 3 times per week for 8 weeks. All treatments were provided by GMB, who developed the protocol. We have described the massage therapy protocol in detail and have shown it to be an effective preventive treatment for the pathologies of repetitive motion disorders,^23,29,33,34,47^ which can be histologically characterized by tissue fibrosis that includes the nerves. The treatment consisted of gentle mobilization, skin rolling, deeper digital pressure, wrist joint play, and whole-limb stretching, designed to emulate treatment provided by massage therapists. As in our previous studies, rats were not anesthetized for the treatment; thus, the treatment forces were limited to levels that did not cause withdrawal or other aversive behavior, but were delivered at the levels of force that were close to the threshold for withdrawal. Descriptive notes were taken regarding the behavior of the rats during the treatment.

With the first group of 16 rats, 2 months following treatments an experienced therapist, HM, palpated the rat’s forelimbs with the goal of determining whether she could discern the irradiated limbs from the control limbs, and the treated limbs from the non-treated limbs. HM’s experience included 14 y treating speech and swallow dysfunction due to head and neck cancer, in a hospital setting, using manual methods for diagnosis and treatment. The palpation study took place over 3 days. HM had not been to this laboratory but was thoroughly familiar with the findings of our preliminary studies, and knew that the outcome assays of this study were focused on deep tissue fibrosis. She had also palpated two rats from the preliminary studies, and stated that she could tell which limbs had been irradiated.

Prior to the experiment, we created a “*fibrosis scale*” that ranged from 0 to 4, with zero being no fibrosis and 4 being severe fibrosis. HM was tasked with stating a number for each limb representing her perception of fibrosis intensity. HM was also asked to describe what she was feeling while GMB took notes. We also created a “*confidence scale*” ranging from 0 to 5, where HM stated her confidence in her decision of which limb had been irradiated (0 being very unconfident, 5 being highly confident).

On the first day, the therapist palpated awake rats with the knowledge of which limbs were irradiated. There were no visual or obvious palpable differences in the rats or their limbs, such as hair loss, erythema, ulceration, atrophy, or volume asymmetry. The only identifying feature of the rats was the ear punch. On the second day, the 16 rats were individually briefly anesthetized by with isoflurane until areflexic to eye touch (4.5–5% induction, 2% maintenance, in pure oxygen). Rats were placed supine by GMB in a random order related to irradiation side. The rat’s ears were completely covered by their heads, with their forelimbs naturally pointing upwards beside their thorax. The therapist was asked to indicate which limb was irradiated based only on palpation (binary choice). Palpation forces were below the force levels that would cause the awake rats to withdraw. On the third day, the therapist was again presented with the anesthetized rats. This time she was told which limb had been irradiated and was tasked with identifying the limbs that had been treated, between groups.

The day after palpation, both limbs of these 16 rats were assayed for indices of tissue fibrosis and inflammation. Rats were deeply anesthetized with isoflurane (5% in pure oxygen). Blood was drawn directly from the heart and set aside. The anesthetized rats were then perfused intracardially with buffered saline followed by 4% paraformaldehyde (PFA). The forelimbs were removed, skinned, and placed in PFA for 24 h. The flexor muscles were removed *en bloc*, moved to 30% sucrose for cryoprotection for 2 d, and placed in 70% ethanol for further processing.

The blood was allowed to clot for 45–60 min and then centrifuged at 4°C for 10 min at 3000 rcf. Serum was pipetted from the tubes and frozen at −30°C until shipped to Eve Technologies (Calgary, Canada) for their Rat Cytokine/Chemokine 27-Plex Discovery Assay (measuring Eotaxin, EGF, Fractalkine, IFNγ, IL-1α, IL-1β, IL-2, IL-4, IL-5, IL-6, IL-10, IL-12p70, IL-13, IL-17A, IL-18, IP-10, GRO/KC, TNFα, G-CSF, GM-CSF, MCP-1, Leptin, LIX, MIP-1α, MIP-2, RANTES, and VEGF-A). All samples were measured in duplicate and the average of the 2 measurements used for comparisons.

Tissue blocks were infused with paraffin, cut to allow transverse and longitudinal sections, and embedded in paraffin for sectioning. Sections were made at 5 µm and mounted on slides. For collagen quantification, sections were stained using Masson’s trichrome or picrosirius red, using standard methods. Other slides were stained for the presence of inflammatory macrophages and myelin degeneration. These slides were blocked with 4% normal goat serum in PBS and reacted with specific antibodies against ED-1 (Serotec, USA; 1:250) or degenerated myelin basic protein (dMBP; recognizes myelin basic protein in demyelinated nerve tissues; AB5864, Millipore-Sigma, Burlington, Massachusetts, United States; 1:500), overnight at 4°C. Slides were washed in PBS, blocked for 30 min in 4% normal goat serum in PBS, and reacted (respectively) with AffiniPure™ Goat Anti-Mouse IgG (H+L) or AffiniPure™ Goat Anti-Rabbit IgG (H+L) (Jackson Labs, USA; 1:500) for 30 min. Product visualization was achieved with the ABC Elite Kit (Vector Labs, USA; USA) for 30 min, followed by DAB (Vector Labs, USA) for ~12 min. Sections were rinsed in distilled water for 10 min, counterstained with H&E, dehydrated with ascending concentrations of alcohol, cleared with xylene, and coverslipped with DPX (Biochemika, Switzerland). Control slides were processed without the primary antibody. The positive control ED-1 was spleen, and the positive control for dMBP was brain.

The second set of 16 rats were primarily used to assay neuropathology, as reported in our model of repetitive motion disorders.^33,38,48^ These rats were irradiated, delivered, housed, and treated with manual therapy using the exact same methods as for the first set of rats.

After 8 weeks, teased fiber electrophysiology was performed on irradiated and untreated limbs using methods detailed previously, performed in the same laboratory and by the same person (GMB).^29,38^ Other rats were terminally anesthetized using >100 mg/kg sodium pentobarbital, and perfused transcardially with buffered saline followed by 4% PFA.

Median nerves were harvested, post-fixed with 4% PFA overnight and then cryoprotected in 30% sucrose. Brain tissue was removed to serve as a positive control for myelin. The nerves and brain were longitudinally sectioned at 10 µm using a cryostat and thaw-mounted onto slides. Subgroups of alternate sections were stained using picrosirius red or processed for the presence of dMBP using immunofluorescence. For immunofluorescence, slides were rinsed in PBS, blocked with 4% normal goat serum in PBS, and reacted overnight with the same antibody to dMBP as described above, at 4°C. On the following day, the slides were again rinsed and blocked, then incubated with Cy™3 AffiniPure Goat Anti-Rabbit IgG (Jackson Labs, USA; 1:100. Brain sections mounted on the same slides were used as positive controls.

### Analysis and statistics

The results of the palpation for the primary outcome were expressed as accuracy (correctly identified irradiated limb/16 total limbs). Descriptive statistics were used for the ordinal measures. For the fibrosis palpation findings, the data were compared using Wilcoxon’s signed rank test.

All rats had one limb irradiated and one limb massaged, and for the inflammatory mediator assays they were grouped into those who had the irradiated limb massaged versus those who did not have the irradiated limb massaged. Serum inflammatory mediator levels were compared between these two groups are presented as graphs and analyzed using Mann–Whitney tests.

Using the trichrome-stained slides, the amount of collagen between muscle fibers, stained blue, was measured to compare radiated and irradiated specimens. The methods here are for each section quantified. First, regions of interest (ROIs) were created that included as much muscle as possible while excluding tendon/ligament, wrinkles/folds, peripheral connective tissue/nerve, extraneous white-space or otherwise non-quantifiable areas (Figure 2a). In some sections, ROIs were selected to exclude from the sample (Figure 2b). The collagen color was thresholded visually based on dye hue, saturation, and intensity. This information was saved as a macro to automate this process and decrease bias. The images were converted to binary images so that collagen showed as white and the background black. The total areas of the selected ROIs were measured. The area of the collagen was measured. The percentage collagen within the section was the area of collagen divided by the area of the combined ROIs.

Nerve collagen content was quantified in the picrosirius-stained slides, using the same method as for the trichrome, except that only one ROI was necessary per slide. Regions of interest were made to avoid obvious artifacts (wrinkles, holes, and folds).

Although the brain sections showed robust labeling with dMBP, immunolabel was not visually present in the nerve sections that were processed on the same slides. Therefore, quantification was not performed.

## Consent to participate

All authors consented to the publication of this article.

## Supplementary Material

03 Supplemental File MOST RECENT.docx

## Data Availability

All data can be accessed by direct contact with GMB by any means.

## References

[1] Purkayastha A, Sharma N, Sarin A, Bhatnagar S, Chakravarty N, Mukundan H, Suhag V, Singh S. Radiation fibrosis syndrome: the evergreen menace of radiation therapy. Asia Pac J Oncol Nurs. 2019;6(3):238–9. doi:10.4103/apjon.apjon_71_18. 31259219 PMC6518980

[2] Yu Z, Xu C, Song B, Zhang S, Chen C, Li C, Zhang S. Tissue fibrosis induced by radiotherapy: current understanding of the molecular mechanisms, diagnosis and therapeutic advances. J Transl Med. 2023;21(1):708. doi:10.1186/s12967-023-04554-0. 37814303 PMC10563272

[3] Fijardo M, Kwan JYY, Bissey PA, Citrin DE, Yip KW, Liu FF. The clinical manifestations and molecular pathogenesis of radiation fibrosis. EBioMedicine. 2024;103:105089. doi:10.1016/j.ebiom.2024.105089. 38579363 PMC11002813

[4] Ridner SH, Dietrich MS, Niermann K, Cmelak A, Mannion K, Murphy B. A prospective study of the lymphedema and fibrosis continuum in patients with head and neck cancer. Lymphat ResearchBiol. 2016;14(4):198–205. doi:10.1089/lrb.2016.0001. PMC5178009 27305456

[5] Harris SR, Tugwell KE. Neurological and dexterity assessments in a woman with radiation-induced brachial plexopathy after breast cancer. Oncologist. 2020;25(10):e1583–e1585. doi:10.1634/theoncologist.2019-0875. 32525604 PMC7543355

[6] Hojan K, Milecki P. Opportunities for rehabilitation of patients with radiation fibrosis syndrome. Rep Pract Oncol Radiother. 2014;19(1):1–6. doi:10.1016/j.rpor.2013.07.007. 24936313 PMC4056465

[7] Delanian S, Lefaix JL. Current management for late normal tissue injury: radiation-induced fibrosis and necrosis. Semin Radiat Oncol. 2007;17(2):99–107. doi:10.1016/j.semradonc.2006.11.006. 17395040

[8] Delanian S, Lefaix JL, Pradat PF. Radiation-induced neuropathy in cancer survivors. Radiother Oncol. 2012;105(3):273–282. doi:10.1016/j.radonc.2012.10.012. 23245644

[9] Choi SH, Hong ZY, Nam JK, Lee H-J, Jang J, Yoo RJ, Lee YJ, Lee CY, Kim KH, Park S, et al. A hypoxia-induced vascular endothelial-to-mesenchymal transition in development of radiation-induced pulmonary fibrosis. Clin Cancer Res. 2015;21(16):3716–3726. doi:10.1158/1078-0432.CCR-14-3193. 25910951

[10] Dombrowsky AC, Schauer J, Sammer M. Acute skin damage and late radiation-induced fibrosis and inflammation in murine ears after high-dose irradiation. Cancers. 2019;11(5). doi:10.3390/cancers11050727. PMC6562452 31130616

[11] Straub JM, New J, Hamilton CD, Lominska C, Shnayder Y, Thomas SM. Radiation-induced fibrosis: mechanisms and implications for therapy. J Cancer Res Clin Oncol. 2015;141(11):1985–1994. doi:10.1007/s00432-015-1974-6. 25910988 PMC4573901

[12] Van Putten JW, Schlosser NJ, Vujaskovic Z, Leest AH, Groen HJ. Superior vena cava obstruction caused by radiation induced venous fibrosis. Thorax. 2000;55(3):245–246. doi:10.1136/thorax.55.3.245. 10679546 PMC1745707

[13] Ferrante MA. Brachial plexopathies: classification, causes, and consequences. Muscle Nerve. 2004;30(5):547–568. doi:10.1002/mus.20131. 15452843

[14] Schierle C, Winograd JM. Radiation-induced brachial plexopathy: review. Complication without a cure. J Reconstr Microsurg. 2004;20(2):149–152. doi:10.1055/s-2004-820771. 15011123

[15] Stoll BA, Andrews JT. Radiation-induced peripheral neuropathy. Br Med J. 1966;1(5491):834–837. doi:10.1136/bmj.1.5491.834. 20790877 PMC1844288

[16] Aggarwal P, Zaveri JS, Goepfert RP, Shi Q, Du XL, Swartz M, Gunn GB, Lai SY, Fuller CD, Hanna EY, et al. Symptom burden associated with late lower cranial neuropathy in long-term oropharyngeal cancer survivors. JAMA Otolaryngol Head Neck Surg. 2018;144(11):1066–1076. doi:10.1001/jamaoto.2018.1791. 30193299 PMC6248190

[17] Dropcho EJ. Neurotoxicity of radiation therapy. Neurol Clin. 2010;28(1):217–234. doi:10.1016/j.ncl.2009.09.008. 19932383

[18] Hutcheson KA, Yuk M, Hubbard R, Gunn GB, Fuller CD, Lai SY, Lin H, Garden AS, Rosenthal DI, Hanna EY, et al. Delayed lower cranial neuropathy after oropharyngeal intensity-modulated radiotherapy: a cohort analysis and literature review. Head Neck. 2017;39(8):1516–1523. doi:10.1002/hed.24789. 28452175 PMC5511776

[19] Cavanagh JB. Effects of x-irradiation on the proliferation of cells in peripheral nerve during Wallerian degeneration in the rat. Br J Radiol. 1968;41(484):275–281. doi:10.1259/0007-1285-41-484-275. 5647982

[20] Zeidman SM, Rossitch EJ, Nashold BS Jr. Dorsal root entry zone lesions in the treatment of pain related to radiation-induced brachial plexopathy. J Spinal Disord. 1993;6(1):44–47. doi:10.1097/00002517-199302000-00008. 8382542

[21] Morales MG, Gutierrez J, Cabello-Verrugio C, Cabrera D, Lipson KE, Goldschmeding R, Brandan E. Reducing CTGF/CCN2 slows down mdx muscle dystrophy and improves cell therapy. Hum Mol Genet. 2013;22(24):4938–4951. doi:10.1093/hmg/ddt352. 23904456

[22] Bechtel W, McGoohan S, Zeisberg EM, Müller GA, Kalbacher H, Salant DJ, Müller CA, Kalluri R, Zeisberg M. Methylation determines fibroblast activation and fibrogenesis in the kidney. Nat Med. 2010;16(5):544–550. doi:10.1038/nm.2135. 20418885 PMC3106179

[23] Barbe MF, Panibatla ST, Harris MY, Amin M, Dorotan JT, Cruz GE, Bove GM. Manual therapy with rest as a treatment for established inflammation and fibrosis in a rat model of repetitive strain injury. Front Physiol. 2021;12:755923. doi:10.3389/fphys.2021.755923. 34803739 PMC8600143

[24] Barbe MF, Hilliard BA, Amin M, Harris MY, Hobson LJ, Cruz GE, Popoff SN. Blocking CTGF/CCN2 reduces established skeletal muscle fibrosis in a rat model of overuse injury. Faseb J. 2020;34(5):6554–6569. doi:10.1096/fj.202000240RR. 32227398 PMC7200299

[25] Barbe MF, Hilliard BA, Amin M, Harris MY, Hobson LJ, Cruz GE, Dorotan JT, Paul RW, Klyne DM, Popoff SN. Blocking CTGF/CCN2 reverses neural fibrosis and sensorimotor declines in a rat model of overuse-induced median mononeuropathy. J Orthop Res. 2020;38(11):2396–2408. doi:10.1002/jor.24709. 32379362 PMC7647961

[26] Barbe MF, Hilliard BA, Delany SP, Iannarone VJ, Harris MY, Amin M, Cruz GE, Barreto‐Cruz Y, Tran N, Day EP, et al. Blocking CCN2 reduces progression of sensorimotor declines and fibrosis in a rat model of chronic repetitive overuse. J Orthop Res. 2019;37(9):2004–2018. doi:10.1002/jor.24337. 31041999 PMC6688947

[27] Fisher PW, Zhao Y, Rico MC, Massicotte VS, Wade CK, Litvin J, Bove GM, Popoff SN, Barbe MF. Increased CCN2, substance P and tissue fibrosis are associated with sensorimotor declines in a rat model of repetitive overuse injury. J Cell Commun Signal. 2015;9(1):37–54. doi:10.1007/s12079-015-0263-0. 25617052 PMC4414846

[28] Fedorczyk JM, Barr AE, Rani S, Gao HG, Amin M, Amin S, Litvin J, Barbe MF. Exposure-dependent increases in IL-1β, substance P, CTGF, and tendinosis in flexor digitorum tendons with upper extremity repetitive strain injury. J Orthop Res. 2010;28(3):298–307. doi:10.1002/jor.20984. 19743505 PMC2807907

[29] Bove GM, Delany SP, Hobson L. Manual therapy prevents onset of nociceptor activity, sensorimotor dysfunction, and neural fibrosis induced by a volitional repetitive task. Pain. 2019;160(3):632–644. doi:10.1097/j.pain.0000000000001443. 30461558 PMC6377318

[30] Bove GM, Harris MY, Zhao H, Barbe MF. Manual therapy as an effective treatment for fibrosis in a rat model of upper extremity overuse injury. J Neurol Sci. 2016;361:168–180. doi:10.1016/j.jns.2015.12.029. 26810536 PMC4729290

[31] Hilliard BA, Amin M, Popoff SN, Barbe MF. Force dependent effects of chronic overuse on fibrosis-related genes and proteins in skeletal muscles. Connect Tissue Res. 2021;62(1):133–149. doi:10.1080/03008207.2020.1828379. 33030055 PMC7718395

[32] Bove GM, Chapelle SL, Hanlon KE, Diamond MP, Mokler DJ, Brakenridge S. Attenuation of postoperative adhesions using a modeled manual therapy. PLOS ONE. 2017;12(6):e0178407. doi:10.1371/journal.pone.0178407. 28574997 PMC5456066

[33] Barbe MF, Harris MY, Cruz GE, Amin M, Billett NM, Dorotan JT, Day EP, Kim SY, Bove GM. Key indicators of repetitive overuse-induced neuromuscular inflammation and fibrosis are prevented by manual therapy in a rat model. BMC Musculoskelet Disord. 2021;22(1):417. doi:10.1186/s12891-021-04270-0. 33952219 PMC8101118

[34] Bove GM, Harris MY, Zhao H, Barbe MF. Manual therapy as an effective treatment for fibrosis in a rat model of upper extremity overuse injury. J Neurological Sci. 2016;361:168–180. doi:10.1016/j.jns.2015.12.029. PMC4729290 26810536

[35] Bove GM, Mokler DJ. Effects of a single dose of psilocybin on cytokines, chemokines and leptin in rat serum. J Psychedelic Stud. 2022;6(3):171–175. doi:10.1556/2054.2022.00230.

[36] Barbe MF, Hilliard BA, Fisher PW, White AR, Delany SP, Iannarone VJ, Harris MY, Amin M, Cruz GE, Popoff SN. Blocking substance P signaling reduces musculotendinous and dermal fibrosis and sensorimotor declines in a rat model of overuse injury. Connect Tissue Res. 2019;61(6):1–16. doi:10.1080/03008207.2019.1653289. PMC7036028 31443618

[37] Zhou Y, Sheng X, Deng F, Wang H, Shen L, Zeng Y, Ni Q, Zhan S, Zhou X. Radiation-induced muscle fibrosis rat model: establishment and valuation. Radiat Oncol (Lond, Engl). 2018;13(1):160. doi:10.1186/s13014-018-1104-0. PMC6114061 30157899

[38] Dilley A, Harris M, Barbe MF, Bove GM. Aberrant neuronal activity in a model of work-related upper limb pain and dysfunction. J Pain. 2022;23(5):852–863. doi:10.1016/j.jpain.2021.12.004. 34958943 PMC9086086

[39] Simpson J, Kelly JP. The effects of isolated and enriched housing conditions on baseline and drug-induced behavioural responses in the male rat. Behavioural Brain Res. 2012;234(2):175–183. doi:10.1016/j.bbr.2012.06.015. 22732260

[40] Dionyssiou D, Nguyen D, Topalis A, Deptula P, Paukshto M, Zaitseva T, Demiri E, Cheva A, Rockson S. Treatment of rat lymphedema by propeller lymphatic tissue flap combined with nanofibrillar collagen scaffolds. J Reconstr Microsurg. 2024;40(2):145–155. doi:10.1055/a-2086-0269. 37142251

[41] McMillan H, Barbon CEA, Cardoso R, Sedory A, Buoy S, Porsche C, Savage K, Mayo L, Hutcheson KA. Manual therapy for patients with radiation-associated trismus after head and neck cancer. JAMA Otolaryngol Head Neck Surg. 2022;148(5):418–425. doi:10.1001/jamaoto.2022.0082. 35297966 PMC8931673

[42] Hartog JM, Warpenburg. Deep friction massage in treatment of radiation-induced fibrosis: rehabilitative care for breast cancer survivors. Intgr Med. 2014;36(5):32–36. doi:10.1097/01.COT.0000454911.02638.d3. PMC4684108 26770116

[43] Bourgeois JF, Gourgou S, Kramar A, Lagarde JM, Guillot B. A randomized, prospective study using the LPG technique in treating radiation-induced skin fibrosis: clinical and profilometric analysis. Skin Res Technol Off J Int Soc Bioeng Skin. 2008;14(1):71–76. doi:10.1111/j.1600-0846.2007.00263.x. 18211604

[44] Barbe MF, Amin M, Harris MY, Panibatla ST, Assari S, Popoff SN, Bove GM. Manual therapy facilitates homeostatic adaptation to bone microstructural declines induced by a rat Model of repetitive forceful task. Int J Mol Sci. 2022;23(12):6586. doi:10.3390/ijms23126586. 35743030 PMC9223642

[45] Du SS, Qiang M, Zeng ZC, Zhou J, Tan Y-S, Zhang Z-Y, Zeng H-Y, Zhong-Shan L. Radiation-induced liver fibrosis is mitigated by gene therapy inhibiting transforming growth factor-β signaling in the rat. Int J Radiat Oncol Biol Phys. 2010;78(5):1513–1523. doi:10.1016/j.ijrobp.2010.06.046. 20932668

[46] Robbins ME, O’Malley Y, Zhao W, Davis CS, Bonsib SM. The role of the tubulointerstitium in radiation-induced renal fibrosis. Radiat Res Mar. 2001;155(3):481–489. doi:10.1667/0033-7587(2001)155[0481:trotti2.0.co;2. 11182800

[47] Bove GM, Chapelle SL, Barrigar MJS, Barbe MF. Manual therapy research methods in animal models, focusing on soft tissues. Front Intgr Neurosci. 2022;15. doi:10.3389/fnint.2021.802378. PMC8834537 35153688

[48] Elliott MB, Barr AE, Clark BD, Amin M, Amin S, Barbe MF. High force reaching task induces widespread inflammation, increased spinal cord neurochemicals and neuropathic pain. Neuroscience. 2009;158(2):922–931. doi:10.1016/j.neuroscience.2008.10.050. 19032977 PMC2661572

