# Supplementary material for: Evaluating massage therapy for radiation-induced fibrosis in rats: preliminary findings and palpation results
Source: Cancer Biol Ther. 2024 Dec 2;25(1):2436694. doi: 10.1080/15384047.2024.2436694 (PMC11622610; doi:10.1080/15384047.2024.2436694)
Supplement: 03 Supplemental File MOST RECENT.docx [file KCBT_A_2436694_SM2290.docx]

**Supplemental File**

**Massage therapy for radiation-induced fibrosis: preliminary findings**


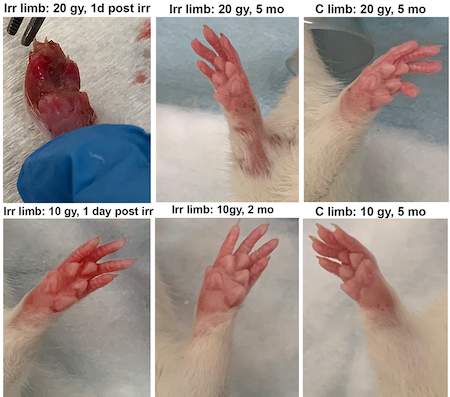
**S1: Appearances following 5 Gray (Gy), 10 Gy, and 20 Gy irradiation.** Anesthetized rats were placed under the lead covers with only their arms extending into the exposure field as described in the main text. The forepaws of 20 Gy irradiated rats showed severe skin burns that largely recovered within 2 weeks, and hair loss in their forearms that recovered after 5 months (above, right, top row). Forepaws of 10 Gy irradiated rats (bottom row) showed transient forepaw skin redness for 2 days, and the health of these rats was otherwise unremarkable following irradiation. Irr = irradiated.

*
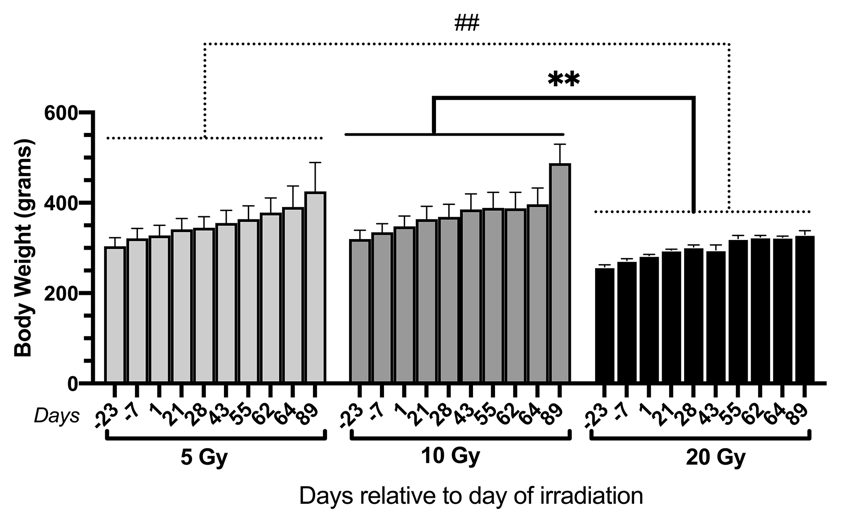
***S2: Weight changes of irradiated rats.** Rats irradiated with 5 or 10 Gy gained body weight normally. Rats irradiated with 20 Gy failed to gain weight across the time shown compared to the other groups (## and ** = p<0.01), suggestive of systemic sickness.

*
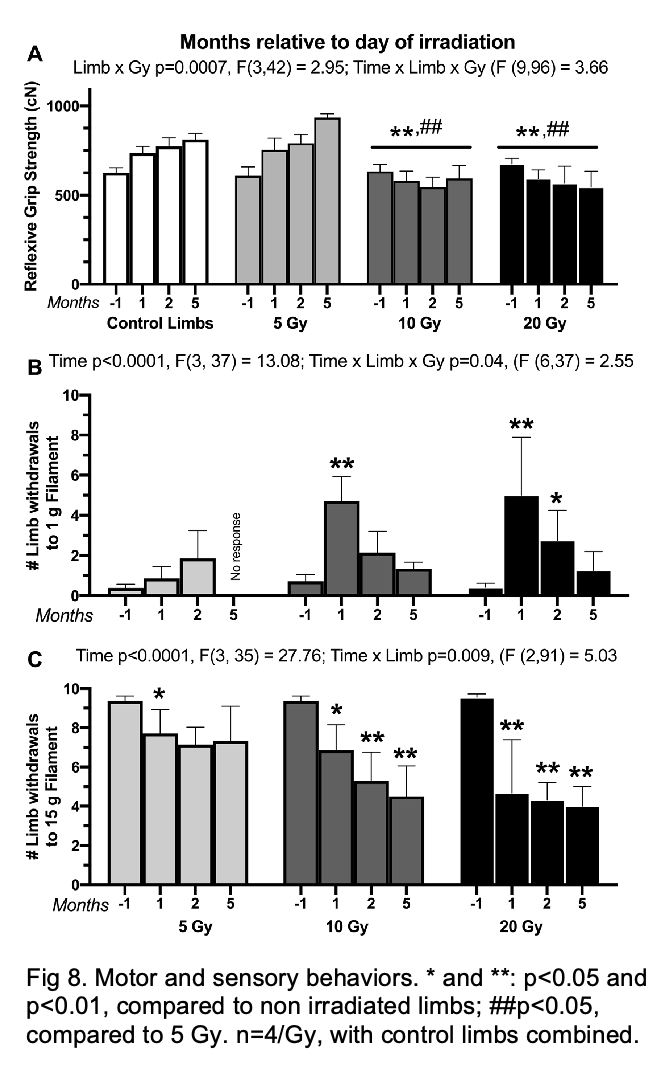
***S3: Motor and sensory behaviors of irradiated rats.** Major complaints in patients who have undergone radiation therapy include distressing paresthesia with progressive losses of touch and thermal perception, pain in some patients, and motor weakness.^1-3^ Longitudinal assays showed that rats irradiated with 10 or 20 Gy doses did not show expected growth related increases in grip strength in their irradiated limb (S3-A), compared to control limbs and 5 Gy rats. Forepaw mechanical sensitivity was tested by probing forepaws with small and large sized monofilaments, with sensitivity indicated by withdrawal of the probed limb. Hypersensitivity of the irradiated forepaws to a 1-gram monofilament was seen at 1-month post-irradiation in 10 Gy rats, and at 1 and 2 months in 20 Gy rats, compared to baseline (S3-B). This is interpreted as allodynia. In contrast, a lowered sensitivity to a 15-gram monofilament was seen in both 10 and 20 Gy rats, compared to baseline levels (S3-C), suggestive of a loss of axons mediating cutaneous sensation. This is consistent with the nerve degeneration observed by histology (see below). * and **: p<0.05 and p<0.01 compared to non-irradiated limbs; ##p<0.05 compared to 5 Gy and control limbs combined.

**S4: Muscle and nerve fibrosis after a single exposure of 5, 10, or 20 Gy.** Since radiation therapy of regions that includes muscles induces similar progressive fibrotic changes in muscle tissues,^4,5^ we examined neuromuscular tissues collected from 5, 10, and 20 Gy exposed animals for evidence of RIF. Post-fixation, tissues were paraffin embedded and sectioned into 5 μm sections (proximal muscle regions) stained with picrosirius red (collagen is stained red), or with Masson’s Trichrome (collagen is stained blue). Figures S4-A-E show clear increases in intramuscular fibrosis in 10 and 20 Gy flexor muscles at 5 months post-irradiation, compared to contralateral non-irradiated control limbs. Sections through the median nerve also showed similar dose-dependent fibrosis (now extraneural and intraneural) in 10 and 20 Gy irradiated rat limbs, as well as increased deposition of collagenous connective tissues around blood vessels (S4-D), similar to prior reports of radiation-induced vascular fibrosis.^6,7^ N=nerve, bv = blood vessel.


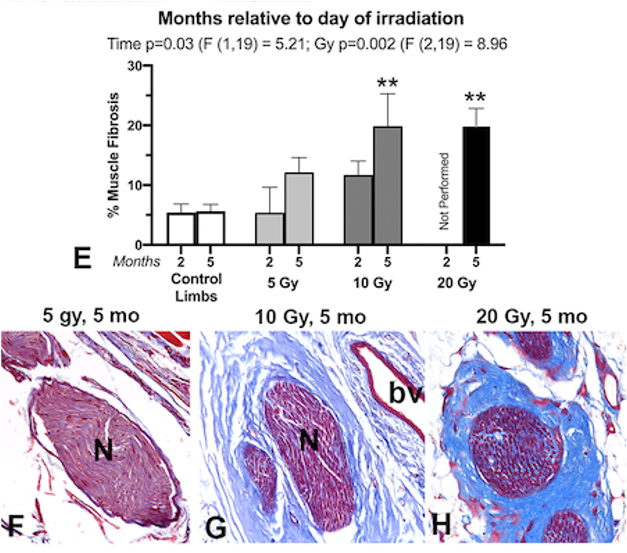

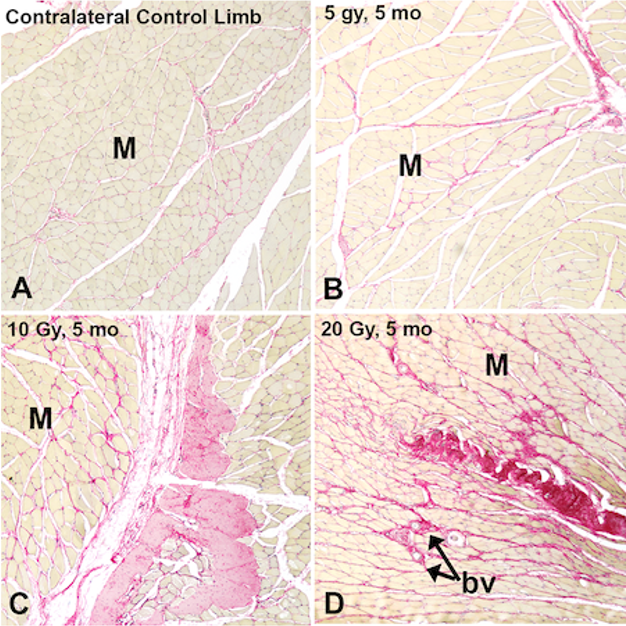


**S5: Electrophysiological signs of neuropathy.**  Radiation therapy is associated with direct and indirect nerve damage.^1,8-12^ Thus, we examined the effects of irradiation on neuronal function using electrophysiological methods developed by Dr. Bove.^13^ A control experiment performed in the laboratory for this project led to recordings from 9 neurons with C-fibers (mean CV = 0.92m/s), of which one had ongoing activity at a rate of 0.15 Hz, and recordings from numerous neurons with faster fibers. There was no ongoing activity or pathological discharge from faster conducting neurons (other than Type II muscle spindles, which normally exhibit irregular ongoing activity). This result was like previous control experiments.^14^ An experiment performed on a rat 2 months after a forelimb was irradiated with 10 Gy yielded recordings from 6 neurons with C-fibers (mean CV = 1.46 m/s), five of which (83%) had slow and irregular ongoing activity (mean 0.44 Hz). Although 2 of 9 faster neurons showed some ongoing activity, it was not deemed neuropathological (i.e., they did not have high rate and or bursting discharge.^14^ This finding is consistent with previous findings during neuritis^15^ and during the active inflammatory phase in rats with overuse injury.^13^ An experiment performed on a rat 5 months after irradiation yielded recordings from 6 neurons with C-fibers (mean CV = 0.85 m/s) and 17 neurons with faster conducting fibers. None had ongoing activity, consistent with control experiments. While not enough data to make a conclusion, it is possible that the nerve changes observed at 2 months healed by 5 months post-irradiation.

**
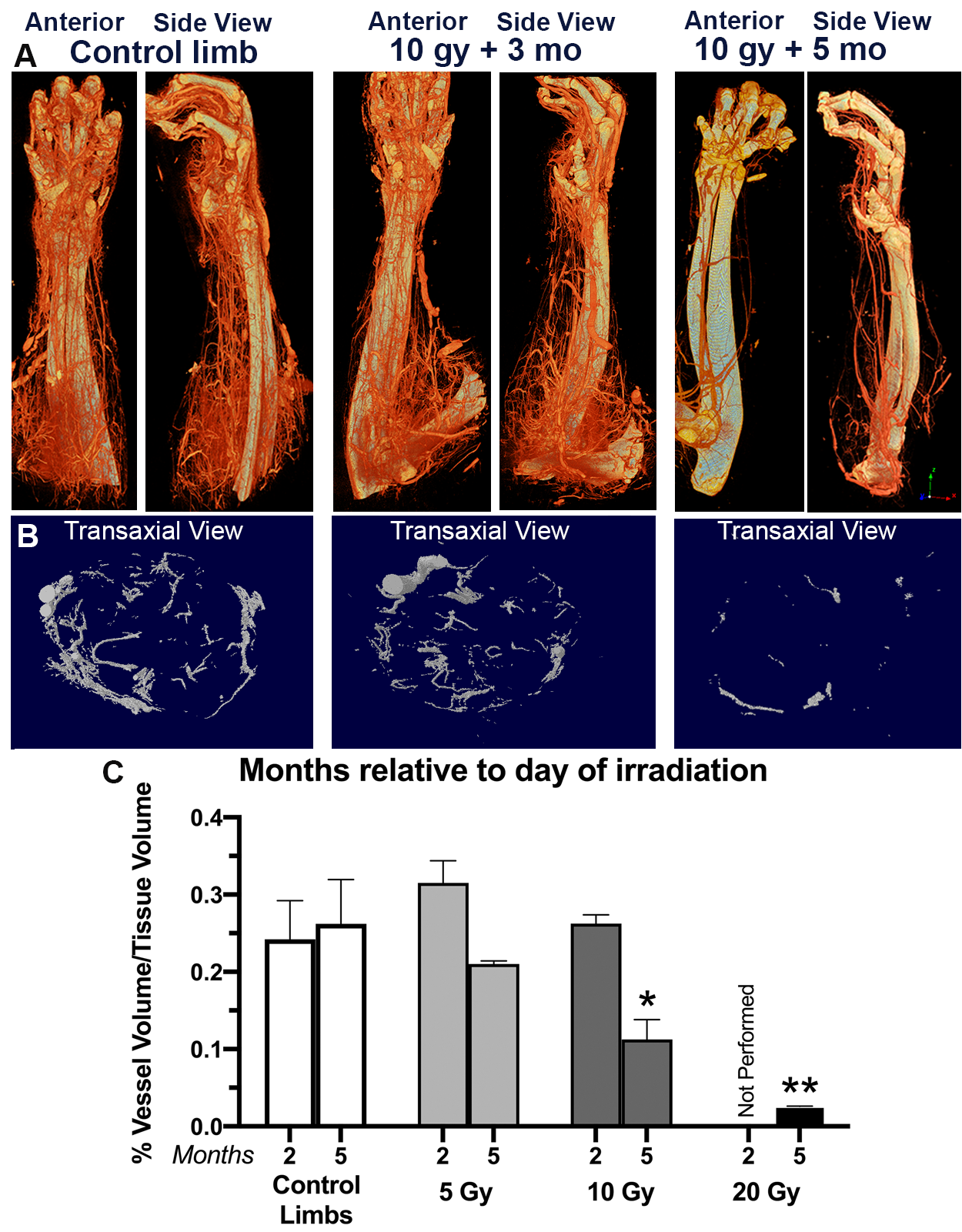
S6: Vascular studies.**  Since vascular injury and capillary network failure have been reported to occur after radiation therapy,^1,12^ additional rats were irradiated as described and used to examine vascular volume. These rats were anesthetized and then perfused intracardially with a radio-opaque polymerizing contrast agent, Microfil (Flow Tech). After polymerization, forearms were scanned in a Micro-CT system, and images compiled into whole forearm 3D models and 2D transaxial 50 “slice” reconstructions of Microfil casted vasculature. Percent vessel volume was calculated using previously described methods.^16^ By 5 months post-irradiation, losses in % vessel volume was observed in 10 and 20 Gy irradiated limbs, compared to contralateral non-irradiated limbs. Although we did not find significant losses in vessels until 5 months after irradiation, as detectable using microCT at the lowest resolution of 3 µm, the significant loss of vessels by 5 months strongly supports the need to investigate earlier indices of endothelial cells damage in irradiation exposed tissues (muscle, tendon, and nerve). * and **: p<0.05 and p<0.01 compared to non-irradiated limbs

**S7: Preliminary findings using manual therapy on the irradiated rats.** We performed manual therapy on rats that had undergone a 10 Gy irradiation of one forearm and compared the results to irradiated rats that did not receive the manual therapy treatment. A. Manual therapy prevented intramuscular collagen formation (red; quantified in B). C. Manual therapy prevented vaculitis as indicated by swollen capillaries (caps). D. Arteriole intimal disruption seen in irradiated rats was not seen in rats that received MMT. E. Fibrosis in the nerve appeared by 1 month of treatment, and this effect lasted without further treatment for 2 months. We found that the manual therapy attenuated several key pathologies, including nerve, muscle, and arterial fibrosis. We also observed what appeared to be nerve vasculitis (swollen capillaries in the nerve) in nerves of untreated 10 Gy, 1-month forearms; a finding not seen in nerves of manual therapy treated rats.


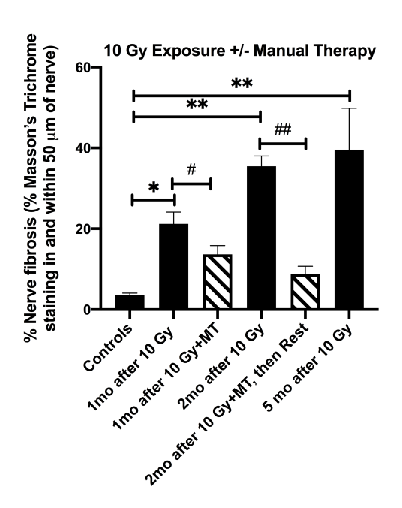

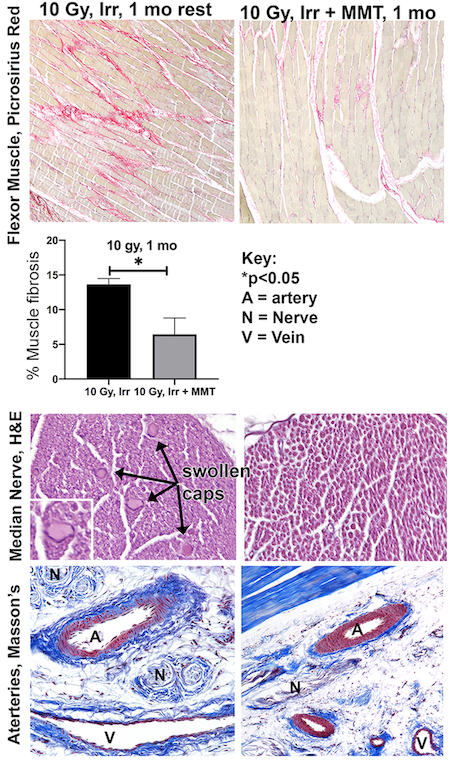


A

B

C

D

E

References

1. Delanian S, Lefaix JL, Pradat PF. Radiation-induced neuropathy in cancer survivors. *Radiother Oncol*. Dec 2012;105(3):273-82. doi:10.1016/j.radonc.2012.10.012

2. Harris SR, Tugwell KE. Neurological and Dexterity Assessments in a Woman with Radiation-Induced Brachial Plexopathy After Breast Cancer. *Oncologist*. Oct 2020;25(10):e1583-e1585. doi:10.1634/theoncologist.2019-0875

3. Hoeller U, Bonacker M, Bajrovic A, Alberti W, Adam G. Radiation-induced plexopathy and fibrosis. Is magnetic resonance imaging the adequate diagnostic tool? *Strahlentherapie und Onkologie : Organ der Deutschen Rontgengesellschaft [et al]*. Oct 2004;180(10):650-4. doi:10.1007/s00066-004-1240-3

4. Zhou Y, Sheng X, Deng F, et al. Radiation-induced muscle fibrosis rat model: establishment and valuation. *Radiation oncology (London, England)*. Aug 29 2018;13(1):160. doi:10.1186/s13014-018-1104-0

5. Horton JA, Hudak KE, Chung EJ, et al. Mesenchymal stem cells inhibit cutaneous radiation-induced fibrosis by suppressing chronic inflammation. *Stem Cells*. Oct 2013;31(10):2231-41. doi:10.1002/stem.1483

6. Takashi I, Ueda Y, Worsdorfer P, Sumita Y, Asahina I, Ergun S. Resident CD34-positive cells contribute to peri-endothelial cells and vascular morphogenesis in salivary gland after irradiation. *J Neural Transm (Vienna)*. Nov 2020;127(11):1467-1479. doi:10.1007/s00702-020-02256-1

7. Sharma UC, Sonkawade SD, Baird A, et al. Effects of a novel peptide Ac-SDKP in radiation-induced coronary endothelial damage and resting myocardial blood flow. *Cardiooncology*. 2018;4doi:10.1186/s40959-018-0034-1

8. Gerard JM, Franck N, Moussa Z, Hildebrand J. Acute ischemic brachial plexus neuropathy following radiation therapy. *Neurology*. Mar 1989;39(3):450-1.

9. Mondrup K, Olsen NK, Pfeiffer P, Rose C. Clinical and electrodiagnostic findings in breast cancer patients with radiation-induced brachial plexus neuropathy. *Acta Neurol Scand*. Feb 1990;81(2):153-8.

10. Cavanagh JB. Effects of x-irradiation on the proliferation of cells in peripheral nerve during Wallerian degeneration in the rat. *Br J Radiol*. Apr 1968;41(484):275-81. doi:10.1259/0007-1285-41-484-275

11. Zeidman SM, Rossitch EJ, Nashold BS, Jr. Dorsal root entry zone lesions in the treatment of pain related to radiation-induced brachial plexopathy. *J Spinal Disord*. Feb 1993;6(1):44-7.

12. Gillette EL, Mahler PA, Powers BE, Gillette SM, Vujaskovic Z. Late radiation injury to muscle and peripheral nerves. *Int J Radiat Oncol Biol Phys*. Mar 30 1995;31(5):1309-18. doi:10.1016/0360-3016(94)00422-H

13. Bove GM, Delany SP, Hobson L, et al. Manual therapy prevents onset of nociceptor activity, sensorimotor dysfunction, and neural fibrosis induced by a volitional repetitive task. *Pain*. Mar 2019;160(3):632-644. doi:10.1097/j.pain.0000000000001443

14. Dilley A, Harris M, Barbe MF, Bove GM. Aberrant Neuronal Activity in a Model of Work-Related Upper Limb Pain and Dysfunction. *J Pain*. Dec 24 2022;23(5):852-863. doi:10.1016/j.jpain.2021.12.004

15. Bove GM, Ransil BJ, Lin HC, Leem JG. Inflammation induces ectopic mechanical sensitivity in axons of nociceptors innervating deep tissues. *J Neurophysiol*. Sep 2003;90(3):1949-55. doi:10.1152/jn.00175.2003

16. Lee S, Barbe MF, Scalia R, Goldfinger LE. Three-dimensional reconstruction of neovasculature in solid tumors and basement membrane matrix using ex vivo X-ray microcomputed tomography. *Microcirculation*. Feb 2014;21(2):159-70. doi:10.1111/micc.12102
